# Metabolic Polymorphisms and Clinical Findings Related to Benzene Poisoning Detected in Exposed Brazilian Gas-Station Workers

**DOI:** 10.3390/ijerph120708434

**Published:** 2015-07-21

**Authors:** Simone Mitri, Antônio Sérgio Almeida Fonseca, Ubirani Barros Otero, Marianne Medeiros Tabalipa, Josino Costa Moreira, Paula de Novaes Sarcinelli

**Affiliations:** 1 Toxicology Laboratory, Center for Studies of Worker’s Health and Human Ecology, Oswaldo Cruz Foundation, Rio de Janeiro 21041-210, Brazil; E-Mails: josinocm@fiocruz.br (J.C.M.); paula@ensp.fiocruz.br (P.N.S.); 2 Medical Ambulatory, Center for Studies of Worker’s Health and Human Ecology, Oswaldo Cruz Foundation, Rio de Janeiro 21041-210, Brazil; E-Mail: antoniosergio@ensp.fiocruz.br; 3 Technical Unit of Occupational Exposure, Environmental and Cancer, Prevention and Surveillance Coordination, National Cancer Institute, Rio de Janeiro 20230-130, Brazil; E-Mails: uotero@inca.gov.br (U.B.O.); mtabalipa@yahoo.com (M.M.T.)

**Keywords:** occupational health, benzene poisoning, gas station worker, genetic polymorphisms, benzene metabolism

## Abstract

Benzene is a ubiquitous environmental pollutant and an important industrial chemical present in both gasoline and motor vehicle emissions. Occupational human exposure to benzene occurs in the petrochemical and petroleum refining industries as well as in gas-station workers, where it can lead to benzene poisoning (BP), but the mechanisms of BP are not completely understood. In Brazil, a significant number of gas-station service workers are employed. The aim of the present study was to evaluate alterations related to BP and metabolic polymorphisms in gas-station service workers exposed to benzene in the city of Rio de Janeiro, Brazil. Occupational exposure was based on clinical findings related to BP, and metabolic polymorphisms in 114 Brazilian gas-station attendants. These workers were divided into No Clinical Findings (NCF) and Clinical Findings (CF) groups. Neutrophil and Mean Corpuscular Volume (MCV) showed a significant difference between the two study groups, and neutrophil has the greatest impact on the alterations suggestive of BP. The clinical findings revealed higher frequencies of symptoms in the CF group, although not all members presented statistical significance. The frequencies of alleles related to risk were higher in the CF group for GSTM1, GSTT1, CYP2E1 7632T > A, but lower for NQO1 and CYP2E1 1053C > T genotypes. Moreover, an association was found between GSTM1 null and alterations related to BP, but we did not observe any effects of other polymorphisms. Variations in benzene metabolizing genes may modify benzene toxicity and should be taken into consideration during risk assessment evaluations.

## 1. Introduction

Benzene is a ubiquitous environmental pollutant, and an important industrial chemical [1,2]. Exposure to it can cause various health hazards and contributes to increasing the risk of blood and bone marrow disorders, including hematotoxicity, genotoxicity and leukemia, even in workers exposed to low levels in the air [3,4,5,6,7]. Exposure to benzene can be environmental or occupational. Benzene is a component of crude oil, cigarette smoke and gasoline [8,9,10,11]. Occupational human exposure to benzene occurs in the petrochemical and petroleum refining industries, as well as from exposure to gasoline and automobile exhaust, so employees of gas-stations are constantly occupationally exposed to higher benzene concentrations and for longer time periods than people driving cars and the general population. In addition, these workers are also exposed to other toxicsubstances present in gasoline, such as toluene, ethylbenzene and xylene [12,13]. This occupation employs a significant number of workers in Brazil, a total of 184,733 distributed over 39,450 gas stations across the country (data of 2010) [14]. 

Occupational exposure to benzene frequently leads to benzene poisoning (BP), and there is an accepted set of signs, symptoms and complications, among which impaired bone marrow is the most important one. The most frequent signs and symptoms are asthenia, myalgia, drowsiness, dizziness and repeated infections, and the most relevant hematological data are neutropenia, leukcopenia, eosinophilia, lymphopenia, monocytopenia, macrocytosis, basophilic stippling, pseudo Pelger and thrombocytopenia [15,16,17]. The mechanisms of BP are not completely understood, but it is generally accepted that it is mediated by a series of benzene activation and detoxification enzymes, which can be modified by genetic variation [18,19,20]. Several genes of benzene metabolism responsible for its activating and detoxifying have polymorphic variants, which may alter its exposure risk [21,22,23,24]. Thus, polymorphism of cytochrome P450 2E1 (CYP2E1), myeloperoxidase (MPO), NAD(P)H: quinoneoxidoreductase 1 (NQO1), glutathione S-transferase theta 1 (GSTT1), and glutathione S-transferase mu 1 (GSTM1), which are involved in benzene activation and detoxification, may affect individual susceptibility to benzene toxicity [20,25,26,27,28,29,30,31,32,33,34]. In the current study, we evaluated alterations related to BP and metabolic polymorphisms in gas station attendants exposed to benzene in the city of Rio de Janeiro, Brazil.

## 2. Material and Methods

### 2.1. Chemicals and Suppliers

Taq DNA polymerase, Taq buffer and MgCl_2_, dNTPs, and digestion enzymes were purchased from Thermo Scientific, (EU) Lithuania. Primers, ladders, and agarose were obtained from Invitrogen by Life Technologies, CA, USA. The Gelred^TM^ staining was supplied by Biotium, Inc., Hayward, CA, USA. Proteinase K was obtained from Roche Diagnostic, Mannheim, Germany.

### 2.2. Study Population and Collection of Samples

A total of 114 workers from seven gas stations in the city of Rio de Janeiro, Brazil, were enrolled in a cross-sectional study. The study was open to participation by male and female workers over 18 years of age. The study was approved by the ethical committee of the Sergio Arouca National School of Public Health (ENSP), Oswaldo Cruz Foundation, number CAAE 0021.0.031.000-10, and all participants signed an informed consent form. The participating workers were interviewed by trained personnel, and a questionnaire was used to obtain general information, including demographic characteristics, cigarette smoking, alcohol consumption, medical history, and occupational data. A blood sample of about 5mLwas collected from each study participant, and these were stored under refrigeration until analysis.

### 2.3. Evaluation of Clinical Alterations Related to Benzene Poisoning

Clinical evaluations were performed at the Center for Studies of Worker Health and Human Ecology (CESTEH) and the National Cancer Institute (INCA), based on the Evaluation Protocols of Health Workers Exposed to Benzene [17,35]. All participants underwent physical and hematological examination. 

Hematological indices such as white blood cell (WBC), erythrocytes, hematocrit, and neutrophil counts were evaluated at INCA. Additionally, data on somatic symptoms were collected from all participants. The interpretation of these data was carried out by physicians from CESTEH. The studied population was categorized into two groups based on clinical findings suggestive of benzene poisoning.

### 2.4. Genotyping

Genotyping was conducted at the Toxicology Laboratory of CESTEH. Genomic DNA was extracted from whole blood samples by the salting-out method. Polymerase Chain Reaction-Restricted Fragment Length Polymorphism (PCR-RFLP) techniques were applied to amplify the polymorphic fragments of CYP2E1 1053C > T and CYP2E1 7632T > A, NQO1 609C > T and MPO 463G > A. Multiplex PCR was done for GSTM1 and GSTT1 deletion polymorphisms, and beta-globin gene was used in the same system as a control. The PCR was done using 50–200 ng of genomic DNA, 0.4 µM of each primer, 1× PCR buffer, 250 µM of dNTPs, 1.5 mM of MgCl_2_ and 1–2.5 units of Taq polymerase in a 50 µL reaction volume. Table 1 shows primers sequences and PCR and digestion conditions for each polymorphism. The resulting products of PCR and digestion were separated on 2–3% agarose gels by electrophoresis and visualized with GelRed^TM^ (Biotium) Nucleic Acid Gel staining and ultraviolet transilluminator. All genotypes were evaluated and independently confirmed by at least two people. A total of 10% of DNA samples were selected randomly for repeat analyses in order to verify the accuracy of the method, and the concordance rate was 100%.

**Table 1:** Primer sequences, amplification and digestion conditions used in the study.

Polymorphisms	Primer sequences	PCR	Digestion	PCR and restrictionpatterns (bp)
CYP2E1 1053C > T	F: 5′-CCAGTCGAGTCTACATTGTCA-3′ R: 5′-TTCATTCTGTCTTCTAACTGG-3′	35 cycles: 95 °C for 1 min, 60 °C for 1 min, and 72 °C for 1 min	*Rsa*I, overnight at 37 °C	PCR:410 CC: 360, 50 CT: 410, 360, 50 TT: 410
CYP2E1 7632T > A	F: 5′-TCGTCAGTTCCTGAAAGCAGG-3′ R: 5′-GAGCTCTGATGCAAGTATCGCA-3′	35 cycles: 94 °C for 30 s; 63 °C for 30 s; 72 °C for 1 min	*Dra*I, 3–5h at 37 °C	PCR: 375 TT:375 TA:375, 249, 126 AA: 249, 126
NQO1 609 C > T	F: 5′-GAGACGCTAGCTCTGAACTGAT-3′ R: 5′-ATTTGAATTCGGGCGTCTGCTG-3′	30 cycles: 94 °C for 10 s; 57 °C for 20 s; 72 °C for 45 s	*Hinf*I, overnight at 37 °C	PCR: 304 CC: 271 CT: 151, 120 TT: 271, 151, 120
MPO 463 G > A	F: 5′-CGGTATAGGCACACAATGGTGAG-3′ R: 5′-GCAATGGTTCAAGCGATTCTT-3′	35 cycles: 91 °C for 1 min; 59 °C for 1 min; 71 °C for 1 min	*Aci*I, 3h at 37 °C	PCR: 350 GG: 169, 120, 61 GA: 289, 169, 120, 61 AA: 289, 61
GSTM1	F: 5′-GAACTCCCTGAAAAGCTAAAGC-3′ R: 5′-GTTGGGCTCAAATATACGGTG-3′	35 cycles: 94 °C for 2 min; 61 °C for 1 min; 72 °C for 2 min		PCR: 215
GSTT1	F: 5′-TTCCTTACTGGTCCTCACATCTC-3′ R: 5′-TCACCGGATCATGGCCAGCA-3′	35 cycles: 94 °C for 2 min; 61 °C for 1 min; 72 °CC for 2 min		PCR: 480
β-globin	F: 5′-CAACTTCATCCACGTTCACC-3′ R: 5′-GAAGAGCCAAGGACAGGTAC-3′	35 cycles: 94 °Cfor 2 min; 61 °C for 1 min; 72 °C for 2 min		PCR: 268

### 2.5. Statistical Analysis

Statistical analysis was carried out using the SPSS statistical software package 17.0.(Chicago, IL, USA). The normality of the distributions was assessed in accordance with the Kolmogorov–Smirnov test. The t-test, χ2-test and the Mann–Whitney U-test were used to analyze differences between the groups. The relationships between several variables, mainly genetic polymorphisms with BP were verified by Spearman correlation analysis. The impacts of genotypes and other analyzed variables on BP were tested using multivariate logistic regression. Deviation from Hardy-Weinberg equilibrium was assessed by a χ2-test. The significance level for all tests was *p* ≤ 0.05.

## 3. Results

### 3.1. Clinical Evaluation and Characteristics of the Studied Population

In accordance with the presence of clinical and laboratory changes that can evolve into BP and with alterations suggestive of BP, subjects were divided into two groups: No Clinical Findings (NCF) and Clinical Findings (CF). From a total of 114 workers, 63.2 % (n = 72) were classified into the CF group, and about 80% of them were male, as shown in Table 2. This table has other information about the workers, such as their basic demographic and occupational characteristics.

**Table 2:** Demographic and occupational characteristics of the study population (114 subjects).

Variables	NCF (n = 42)	CF (n = 72)
	n (%)	n (%)
Sex		
Male	35 (83.3)	52 (72.2)
Female	7 (16.7)	20 (27.8)
		
Ethnic background/Skin color		
White	13 (33.3)	27 (39.1)
Black	6 (15.4)	6 (8.7)
Mulatto	17 (43.6)	33 (47.8)
Asian	2 (5.1)	1 (1.4)
Indigenous	1 (2.6)	2 (2.9)
		
Education level		
Illiterate	-	1 (1.5)
Elementary school	11 (28.9)	24 (35.3)
Middle school	11 (28.9)	24 (25.3)
High school	16 (42.2)	18 (26.5)
>High school	-	1 (1.5)
		
Family income (R$)		
Mean	1,483.14 ± 114.38	1,558.22 ± 111.82
Min	600.00	500.00
Max	3,500.00	6,000.00
		
Age (years)		
Mean	35	38.4
Min	20	19
Max	61	82
		
Smoking consumption		
Yes	10 (24,4)	13 (18,3)
No	31 (75,6)	58 (81,7)
		
Alcohol consumption		
Yes	27 (65.9)	55 (77.5)
No	14 (34.1)	16 (22.2)
		
Exposure duration (years)		
Mean	13 ± 1.97	15.2 ± 1.38
Min	-	-
Max	42	52

The data presented in Table 3 represent the differences in hematological indices between the groups. The CF group showed lower values than NCF, except to lymphocytes and Mean corpuscular volume (MCV). We found an association between neutrophil and BP (OR= 1.130, 95% CI= 1.035–1.234).

**Table 3:** Hematological values observed in NCF and CF.

Variables	NCF Mean ± SD	CF Mean ± SD	*p-value*
Red Blood Cell (million/mL)	4.90 (±0.3318)	4.77 (±0.5513)	0.139
Hemoglobin(g/dL)	14.02 (±1.195)	14.00 (±1.698)	0.940
Hematocrit (%)	41.33 (±3.070)	41.00 (±4.595)	0.673
MCV (fL)	84.49 ±0.63	86.13 ± 0.80	0.005
WBC (cells/µL)	7,830 (±2,115)	7,260 (±1,628)	0.125
Neutrophil (%)	58.53 (±5.883)	53.97 (±9.537)	0.003
Lymphocyte (%)	30.93 (±5.555)	34.20 (±8.757)	0.019
Platelet (billion/L)	252.10 (±57.901)	243.52 (±63.108)	0.487

**Figure 1:**
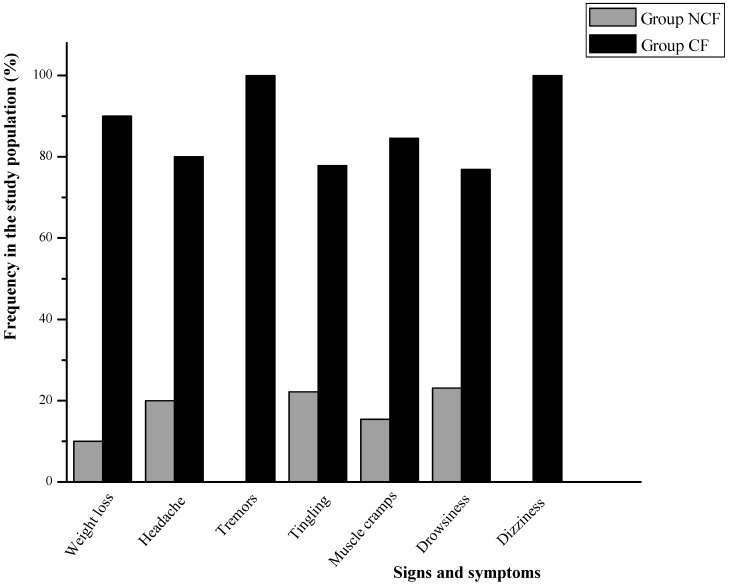
Comparison of somatic symptoms of BP between groups (*SD, weight loss: ±0.15 (NCF) and ±0.33 (CF); headache: ±0.47 (NCF) and ±0.49 (CF); tremors: ±1.39 (NCF) and ±0.26 (CF); tingling: muscle cramps: ±0.28 (NCF) and ±0.47 (CF); drowsiness: ± 0.40 (NCF) and ± 0.35 (CF); dizziness: ±0.26 (NCF) and ±0.33 (CF).

The principal somatic symptoms reported by the subjects are illustrated in Figure 1.The graph shows that the CF group recorded higher frequencies of headaches (80% *vs.* 20%; *p* = 0.023), muscle cramps (84.6% *vs.* 15.4%; *p* = 0.009), tingling (77.8% *vs.* 22.2%; *p* = 0.161), drowsiness (76.9% *vs.* 23.1%; *p* = 0.243), dizziness (75% *vs.* 25%; *p* = 0.369), weight loss (90% *vs*. 10%; borderline *p* = 0.065), and recurrent infections (0 *vs*. 100%; *p* = 0.037). 

### 3.2. Genetic Analysis

The following genotype frequencies of each polymorphism in the population were found:GSTM1-68.7% (positive) and 31.3% (null); GSTT1-75.8% (positive) and 24.2% (null); NQO1-64.1% (TC), 30.4% (TC), 5.4% (TT); MPO-43.2% (GG), 49.5% (GA), 7.4% (AA); CYP2E1 *Rsa*I-77% (CC),13.8% (CT), 9.2% (TT) and; CYP2E1 *Dra*I-82.1% (TT), 16.8% (TA), and 1.1% (AA).

The variant allele frequencies among NCF and CF groups are summarized in Table 4. The CF group presented higher frequencies of variant for CYP2E1 7632T > A; null alleles for GSTM1 and GSTT1. However, although there were lower frequencies for NQO1 and CYP2E1 1053C > T than in the NCF group, there was no statistical difference between them. For GSTM1, the frequency null genotype was 0.26 and 0.34 for the NCF and the CF groups, respectively. In regard to the GSTT1 gene, the groups presented frequencies of 0.20 (NCF) and 0.26 (CF). The T allele of CYP2E1 1053C > T showed 0.21 (14.3% homozygous and 14.3% heterozygous) and 0.14 (6.8% homozygous and 13.6% heterozygous) for NCF and CF, respectively. For CYP2E1 7632T > A were found 0.06 (12.1% of heterozygous and no one homozygous) in NCF, and 0.11 (19.4% heterozygous and 1.6% homozygous) in CF. NQO1 showed 0.32 (38.7% of heterozygous and 6.5% of homozygous) in NCF and 0.18 (26.2% heterozygous and 4.9% homozygous) in CF. MPO polymorphism, whose variant allele is not related to susceptibility but to protection, presented 0.30 (48.5% heterozygous and 9.1% homozygous) in NCF, and 0.31 (50% heterozygous and 6.5% homozygous) in CF group. The genotype distributions were in accordance with Hardy-Weinberg, except for GSTM1,GSTT1 and CYP2E1 1053C > T.

**Table 4:** Variant allele frequencies in NCF and CF.

Polymorphisms	Variant allele	NCF	CF	*p*-value
*NQO1*	T^a^	0.32	0.18	0.233
CYP2E1 1053C > T	C^a^	0.21	0.14	0.394
CYP2E17632T > A	A^a^	0.06	0.11	0.284
GSTM1	null^a^	0.26	0.34	0.452
GSTT1	null^a^	0.20	0.26	0.539
MPO	A^b^	0.30	0.31	0.639

^a^ alleles related to risk; ^b^ alleles related to protection.

According to the literature, the alleles presented in Table 4 are related to benzene exposure and its effects on health [4,19,21,22,28,29]. The number of alleles related to risk showed associations with the presence of clinical findings of BP (Spearman Chi-Square = 0.196; *p*-value = 0.024). Logistic regression showed that GSTM1 null genotype had an impact on the changes related to BP (OR = 5.131, 95% CI = 1.137–23.151). No significant influence of the other polymorphisms was observed in the changes related to BP (results not shown).

## 4. Discussion

We evaluated alterations related to BP and benzene metabolic polymorphisms in gas-station workers in Rio de Janeiro, Brazil since this professional category has been occupationally exposed to benzene. Studies have shown that gas-station service workers are exposed to levels of tens to hundreds of ppb [14,36,37]. However, there have been few studies carried out of individual genetic susceptibility in gas-station workers. 

In the present study, most of the workers showed clinical and laboratory changes that can evolve into BP, 63.2%. Diagnosis of BP from occupational exposure is predominantly clinical and epidemiological, based on medical history, laboratory data and clinical symptoms [17]. BP is considered when the individual presents a set of signs and symptoms after exposure to benzene. Common symptoms are asthenia, headache, myalgia, drowsiness, dizziness, tremor and recurrent infections [15,16,38], and the most relevant hematological data are a decrease in blood cells and macrocytosis [39,16,40,41]. 

We found that although the difference was not significant, the CF group showed a reduced blood cell count in comparison with the NCF one. Neutrophil and MCV (indicative of macrocytosis) showed a significant difference between groups, and the percentage of neutrophil was the hematological variable with the greatest impact on the alterations suggestive of BP. These results were consistent with preview studies. The study of Qu*et al*. (2002) [42] observed a decrease in red and white cell counts, especially neutrophils, in Chinese workers exposed to benzene. Lan *et al*. (2004) [4] also found a decrease in almost all blood cells in an occupationally exposed population, which highlights that the broad action of benzene on various subtypes of blood cells seems to provide strong evidence of its toxicity in the progenitor cells of the bone marrow cells. 

In relation to symptoms, we observed higher frequencies of headaches, recurrent infections, muscle cramps, tingling, drowsiness, dizziness and weight loss in the CF group, although not all with statistical significance. D’Alascio *et al*. (2014) [38] reported similar symptoms in Brazilian gas-station workers with a higher prevalence of fatigue, headache, tremor, insomnia, and drowsiness. 

Six polymorphisms in benzene metabolizing genes were analyzed to verify the presence of genetic susceptibility in these workers. The frequencies of alleles related to risk were higher in the CF group for GSTM1, GSTT1, CYP2E1 7632T > A, but lower for NQO1 and CYP2E1 1053C > T polymorphisms. The MPO variant allele showed no difference. Except for GSTM1 and CYP2E1 1053C > T, the allele frequencies were similar to the findings of most other studies [43,44,45,46,47,48,49,50]. Chen *et al*. (2007) [28] investigated the relationship between the same polymorphisms and BP in workers exposed to benzene, and they found an association with NQO1 and GSTT1, but not with CYP2E1, GSTM1, and MPO. Study of Wan *et al*. (2002) [22] also found association between GSTT1 null genotype and significant increase in risk of BP, however the studies of Sun *et al*. (2008) [51] and Lan *et al*. (2004) [4] reported no association. We did not observe association between clinical findings related to BP and NQO1, MPO, CYP2E1 and GSTT1 polymorphisms, but we found an association with GSTM1. 

The literature also has reported the influence of gene–gene interaction on benzene toxicity. Several studies have shown that combinations of high CYP2E1 and MPO activities and low or negligible NQO1, GSTM1 and GSTT1 activities (high bioactivation combined with low detoxification), may increase the risk of benzene-induced toxicity [12,28,32,52,53,54,55,56]. We observed an association between subjects carrying more alleles related to risk and clinical changes related to benzene poisoning, which may suggest that interactions of multi-genes in benzene metabolism may contribute to the development of these changes.

In conclusion, our findings suggest that the GSTM1 null genotype may play a role in the development of clinical alterations related to BP. However, further studies with larger sample sizes will be needed to confirm these findings. Although the literature data on genetic susceptibility to BP have so far been inconclusive, it is generally accepted that variations in benzene metabolizing genes may modify benzene toxicity and should be taken into consideration when carrying out risk assessments for exposed workers.
